# Facebook as a Novel Tool for Continuous Professional Education on Dementia: Pilot Randomized Controlled Trial

**DOI:** 10.2196/16772

**Published:** 2020-06-02

**Authors:** Windy SY Chan, Angela YM Leung

**Affiliations:** 1 School of Health Sciences Caritas Institute of Higher Education New Territories China (Hong Kong); 2 Faculty of Health and Social Sciences The Hong Kong Polytechnic University Kowloon China (Hong Kong); 3 Centre for Gerontological Nursing School of Nursing The Hong Kong Polytechnic University Kowloon China (Hong Kong)

**Keywords:** dementia, Facebook, social network sites, continuous professional education

## Abstract

**Background:**

Social network sites (SNSs) are widely exploited in health education and communication by the general public, including patients with various conditions. Nevertheless, there is an absence of evidence evaluating SNSs in connecting health professionals for professional purposes.

**Objective:**

This pilot randomized controlled trial was designed to evaluate the feasibility of an intervention aiming to investigate the effects of a continuous professional education program utilizing Facebook to obtain knowledge on dementia and care for patients with dementia.

**Methods:**

Eighty health professionals from Hong Kong were recruited for participation in the study and randomized at a 1:1 ratio by a block randomization method to the intervention group (n=40) and control group (n=40). The intervention was an 8-week educational program developed to deliver updated knowledge on dementia care from a multidisciplinary perspective, either by Facebook (intervention group) or by email (control group) from October 2018 to January 2019. The primary outcomes were the effects of the intervention, measured by differences in the means of changes in pre- and postintervention scores of knowledge assessments from the 25-item Dementia Knowledge Assessment Scale (DKAS) and formative evaluation of 20 multiple choice questions. Other outcome measurements included participant compliance, participant engagement in Facebook, satisfaction, and self-perceived uses of Facebook for continuing professional education programs.

**Results:**

Significantly more intervention group participants (n=35) completed the study than the control group (n=25) (*P*<.001). The overall retention rate was 75% (60/80). The mean of changes in scores in the intervention group were significant in all assessments (*P*<.001). A significant difference in the mean of changes in scores between the two groups was identified in the DKAS subscale Communication and Behavior (95% CI 0.4-3.3, *P*=.02). There was no significant difference in the total DKAS scores, scores of other DKAS subscales, and multiple choice questions. Participant compliance was significantly higher in the intervention group than in the control group (*P*<.001). The mean numbers of participants accessing the learning materials were 31.5 (SD 3.9) and 17.6 (SD 5.2) in the intervention and control group, respectively. Polls attracted the highest level of participant engagement, followed by videos. Intervention group participants scored significantly higher in favoring the use of Facebook for the continuing education program (*P*=.03). Overall, participants were satisfied with the interventions (mean score 4 of a total of 5, SD 0.6).

**Conclusions:**

The significantly higher retention rate, together with the high levels of participant compliance and engagement, demonstrate that Facebook is a promising tool for professional education. Education delivered through Facebook was significantly more effective at improving participants’ knowledge of how people with dementia communicate and behave. Participants demonstrated positive attitudes toward utilizing Facebook for professional learning. These findings provide evidence for the feasibility of using Facebook as an intervention delivery tool in a manner that can be rolled out into practical settings.

## Introduction

### Background

Social network sites (SNSs) have become an essential part of the daily lives of billions of users globally. Facebook currently represents the largest social network in the world, which engages more than 2.45 billion users globally [1]. SNSs are widely exploited in research on health behavior-related outcomes and health communication by both the general public and patients [2,3]. A systematic review showed that health professionals have also extensively adopted SNSs for professional purposes [4]. SNSs are used to support the delivery of clinical services, make referrals, and share information [5-7], demonstrating benefits in network building and professional collaboration [8-10]. Facebook is a novel tool for enhancing educational interactions [4,11], sharing domain knowledge through developing professional communities [12,13], and staying abreast of news and information pertaining to professional needs and interests [14-16]. With increased adoption of SNSs, it is critical to evaluate the effectiveness of such utilization for professional purposes by health professionals. This pilot study is the first to evaluate the effects SNSs in continuous professional education (CPE) programs by utilizing Facebook in the delivery of education on dementia care to a cohort of health professionals.

### Need for CPE on Dementia

Dementia has been acknowledged as a significant global public health issue [17,18]. In 2015, it was estimated that 46.8 million people were living with dementia worldwide, with those numbers expected to almost double every 20 years, reaching up to 131.5 million by 2050 [19]. Timely detection and proper management of dementia are critical to allow patients to plan for their future care while they still have the capacity to make important decisions [20]. However, inadequate effort has been made to address the continuous educational needs of the health care workforce on the topic of dementia [21]. Low levels of dementia knowledge among health care personnel remain common [21], representing a major barrier to providing appropriate end-of-life care to people with dementia [22]. The limited teaching hours in preregistration programs was identified as one of the main factors associated with the lack of knowledge of dementia [23]. Postregistration training on dementia care has been offered in some programs, but the most common approach was traditional small to large group face-to-face delivery [21], which has limitations in terms of resources, time, and geographical factors. Electronic learning (eLearning) options for CPE programs offer flexibility with respect to both the time and place of learning. Nevertheless, these types of programs can be accompanied by inadequate levels of participant interaction, reflection, practice, and application to practice [24,25]. With the evolving utilization of SNSs by health professionals, there is potential to make the most of SNSs to meet professional education requirements and promote interaction while avoiding a significant resource burden.

Although some research has been conducted to investigate the uses of SNSs in health communication and education among the general public and patients, there is a general lack of literature on the topic, with a notable sparsity of evidence from intervention trials on evaluating SNSs in connecting health professionals for professional communication [4]. The findings obtained to date are in line with reviews on professional dementia education, in which most of the included studies used weak research designs that were unable to identify the direct effects of the interventions [26,27].

### Aim and Objectives

This pilot trial was designed to fill the gaps in the current literature, representing the first pilot randomized controlled trial (RCT) examining the effects of an SNS on professional education by health professionals. The aim of the study was to evaluate the feasibility of an interventional trial designed to investigate the effects of a CPE program utilizing Facebook on dementia knowledge and care. The objectives to achieve the research aim were to: (1) quantitatively evaluate the effects of a Facebook intervention by measuring the change in knowledge pre and postintervention, (2) quantitatively evaluate the feasibility and acceptability of the intervention, and (3) qualitatively assess participant perceptions of the intervention related to recruitment, delivery, and usability.

## Methods

### Overview

The study was a pilot randomized, unblinded, controlled trial with mixed-research methods (quantitative and qualitative). The intervention was an 8-week CPE program on dementia delivered to participants either by Facebook (intervention group) or by email communications (control group) from October 2018 to January 2019. Ethical approvals were obtained from the Hong Kong Polytechnic University (reference number: HSEARS20180307007) and the Caritas Institute of Higher Education (IRG180100). Trial reporting follows the CONsolidated Standards of Reporting Trials (CONSORT) 2010 statement: extension to randomized pilot and feasibility trials [28].

### Subject Recruitment and Randomization

Health professionals were recruited via promotion of the trial at professional conferences, and by invitations disseminated through professional bodies and health institutes. Inclusion criteria were being a health professional, having access to the internet, in addition to having an email account and Facebook account or willing to create one otherwise. Individuals were excluded if they were working at institutes specializing in the delivery of dementia or geriatric care or had prior training in dementia care that was completed in the past 6 months.

Before randomization, participants were required to complete baseline assessments, knowledge tests, and consent forms. In addition to collecting background information such as age, gender, occupation, and years of working experience, the baseline assessment also surveyed the participants’ habits with respect to the use of SNSs, including SNS choices and frequency of use.

Consenting participants were randomized to two groups at a 1:1 ratio by a block randomization method in view of the small sample size [29]. The randomization was performed by an independent researcher who had no involvement in the trial. The block size was blinded from the investigator performing this study.

### Intervention

The learning materials (ie, the educational intervention) were developed by a team of experts from the relevant disciplines, including medicine, nursing, pharmacy, occupational therapy, speech therapy, and social sciences, to provide updated interdisciplinary knowledge on dementia care. Upon completion, participants would be able to (1) describe the prevalence, pathophysiology, and recognition of dementia; (2) apply the key screening and assessment tools for diagnosing dementia; (3) explain management and support to both elderly patients with dementia and their caregivers; and (4) appraise their roles in the provision of dementia care.

Along with the textual content, a collection of multimedia materials was selected to supplement participant learning, including websites, educational videos, pictures, diagrams, and interactive online games.

Participants in both groups received the same content and were expected to spend approximately 15 minutes daily reading the materials. Together with the time spent in assessments, the total learning hours amounted to approximately 12 hours throughout the 8-week intervention period.

**Figure 1:**
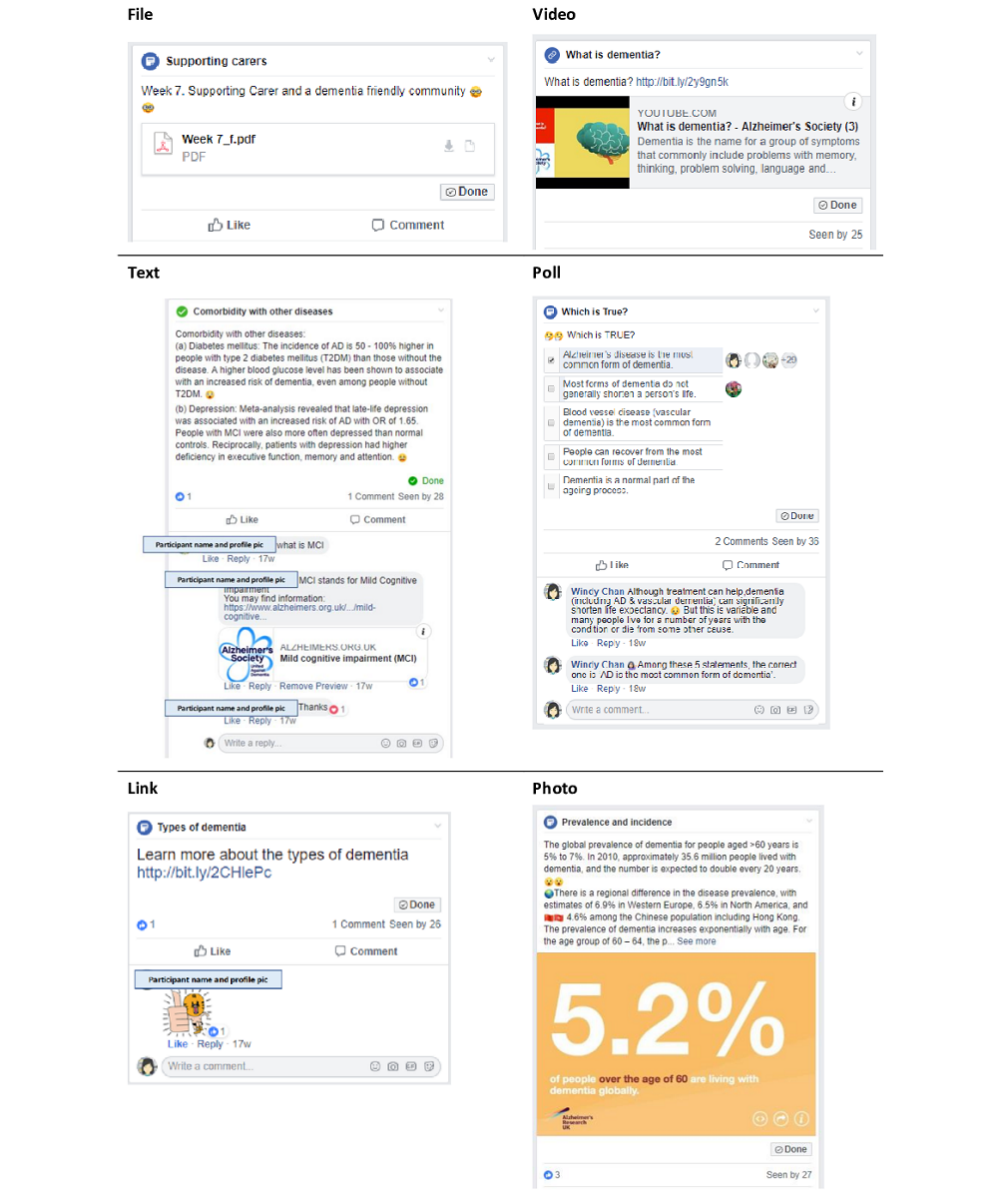
Representative screenshots of six types of Facebook posts.

In the intervention group, a Facebook Social Learning Group was created to deliver the CPE program. A Social Learning Group has additional features to an ordinary Facebook group, which facilitate the retrieval of learning materials and tracking participant engagement. The learning materials were uploaded every 1 to 3 days in the form of Facebook posts, photos, videos, text, files, or polls (Figure 1). A variety of components and post lengths were utilized, with the aim of maximizing participant engagement and interest [30,31]. There were typically 14 posts per week, including a post containing the notes (learning materials) of the week in PDF format. Participants were encouraged to comment, react, and interact with the other group members but were not allowed to create a new post.

In the control group, before the beginning of each study week, participants received emails containing their individual Bitly links to the PDF notes of that week. Bitly is a link management platform that enables analysis of the activity of each link [32]. Contents of notes were identical to those received by the intervention group participants, including the revision questions, photos, videos, games, and websites. Control group participants could access the multimedia content using the Bitly links inserted in the PDF notes, but could not take part in the polls or view the previews of links generated by Facebook. During the intervention period, control group participants received a total of 8 sets of notes.

### Outcome Measures

#### Primary Outcome: Quantitative Evaluation of the Effects of a Facebook Intervention

The effectiveness of using Facebook to deliver the CPE program was measured by the knowledge gain between the pre and postintervention knowledge tests, calculated as the difference in scores. The knowledge gain in the two groups was also compared.

The knowledge tests consisted of formative evaluation, 20 multiple choice questions, and the 25-item Dementia Knowledge Assessment Scale (DKAS). Content for the multiple choice questions was drawn directly from the learning materials throughout the 8 learning weeks. The DKAS has been validated as a reliable measure of dementia knowledge for a wide spectrum of populations, including health professionals. The DKAS elucidates respondents’ knowledge characteristics across four coherent domains: Causes and Characteristics, Communication and Behavior, Care Considerations, and Risks and Health Promotion [33]. A pilot assessment of the knowledge tests was performed to determine the coverage of questions, level of difficulty, and potential ceiling effects.

#### Quantitative Evaluation of Participant Compliance

Participant compliance was quantified by the number of week(s) (out of 8) the material was accessed by the participants, recorded by the “Seen by” feature of Facebook in the intervention group and by the Bitly link in the control group.

In Facebook, the “Seen by” feature of a post automatically records the individual group members that had seen the post (Figure 1). In the control group, each participant had their own 8 unique Bitly links to access the corresponding 8 weeks of materials. A participant clicking on the Bitly link was counted as having accessed the materials for that week. Bitly links were also created to track participant compliance in accessing the recommended external learning resources such as videos and websites. 

#### Quantitative Evaluation of Intervention Group Participant Engagement

In studying the communication artifacts occurring in Facebook, engagement was defined as the metric of actions that participants took in the group during the intervention period. In addition to ordinary actions such as comment and reactions, a unique feature of the Facebook Social Learning Group is the “Done” button (Table 1). Participants were encouraged to click the “Done” button to share their learning progress. A set of descriptive data was also extracted from analyzing participant engagement in the intervention group, considering features that have been generally used in previous Facebook-based studies [30,31].

**Table 1:** Participant engagement actions on Facebook.

Engagement Action	Definition
Comment	A participant commented on a post or a comment made by another member in the group
React	A participant clicked the reaction button on a post to share different reactions: Like, Love, Haha, Wow, Sad, or Angry
Done	A participant clicked the “Done” button of a post. The “Done” button will turn green after being clicked.

#### Quantitative Evaluation of Participant Satisfaction

All participants were invited to complete a satisfaction evaluation form developed for the effective appraisal of training delivery for health professionals [34]. The form contained 10 5-point Likert-style positively phrased statements focusing on the achievement of program objectives, impact on learning, learning experiences, and application to clinical practice. There were 3 questions that evaluated the program length, information quantity, and difficulty level. A pilot assessment of the evaluation form was performed to determine the coverage of questions.

#### Quantitative Evaluation of Participants’ Attitudes Toward Using Facebook for the CPE Program

Participants’ attitudes on the professional use of Facebook were evaluated in a questionnaire modified from the Continuous Medical Education App Attitudes Survey Instrument, a validated measure of participant attitudes toward mobile app use in a continuous medical education program [35]. There were 10 5-point Likert-style positively phrased statements evaluating the educational value, acceptability, usability, and future feasibility of using Facebook to deliver the program. A pilot assessment of the survey was performed to determine the question coverage.

#### Qualitative Assessment of Feasibility and Acceptability

To investigate the pilot study’s feasibility and acceptability, all participants were invited to complete a feedback form on their learning experiences and to take part in short semistructured interviews. To encourage participation of interviews, reminders were sent twice by Facebook messages (intervention group) and emails (control group).

In the feedback form, qualitative data were obtained via two free-form questions that asked the participants about their perceptions of the most valuable sections as well as suggestions for improvement.

The semistructured interviews were conducted following a flexible interview guide to explore a wider range of respondents’ perceptions and comments. Participants were invited to share their views on (1) promotion, recruitment, and randomization arrangement; (2) delivery of the educational intervention; (3) usability of the learning materials; (4) feasibility and acceptability of the study; and (5) comments for further improvement. Respondents could refuse to answer any question or ask questions. Interviews were audio-recorded and transcribed manually. Data were organized using a Microsoft Excel 2016 spreadsheet.

Thematic analyses of the contents of feedback forms and semistructured interviews were conducted independently by the authors. Discrepancies on data analyses, coding, and themes generated were resolved by discussion until consensus was reached [36].

### Statistical Analysis

A literature search did not identify any RCTs conducted on the effects of an educational intervention utilizing SNSs that could be used as a basis for calculating sample size. This study conservatively assumed a small effect size of 0.2. For a full RCT designed with 90% power and a two-sided 5% significance threshold, the recommended sample size for a pilot trial is 30 per group [37]. Assuming a dropout rate of 10%, the total target number of participants was 66. Slightly more participants (N=80) were recruited for this study due to positive responses from professional bodies.

Participant demographics and SNS usage characteristics were treated as categorical variables. The Chi square test and Fisher exact test were used to analyze dichotomous and categorical variables. Participant performance on the knowledge test is presented descriptively. To compare the knowledge gains of the two groups (primary outcome), an independent sample *t* test was used. The effect size of the outcome measure between the two groups was estimated according to the formula proposed by Morris [38] for mean pre-post changes between the intervention and control groups. An intention-to-treat approach was adopted in this study. Scores in the evaluation and survey on the use of Facebook (secondary outcome) are presented as descriptive statistics and were analyzed by a two-sample *t* test or Fisher exact test. *P*≤.05 was considered significant. Statistical analyses were conducted using R v. 3.1.2 (R Foundation for Statistical Computing, Vienna, Austria) and IBM SPSS Statistics version 23 (IBM Corp, Armonk, NY, USA).

## Results

### Participants

**Figure 2:**
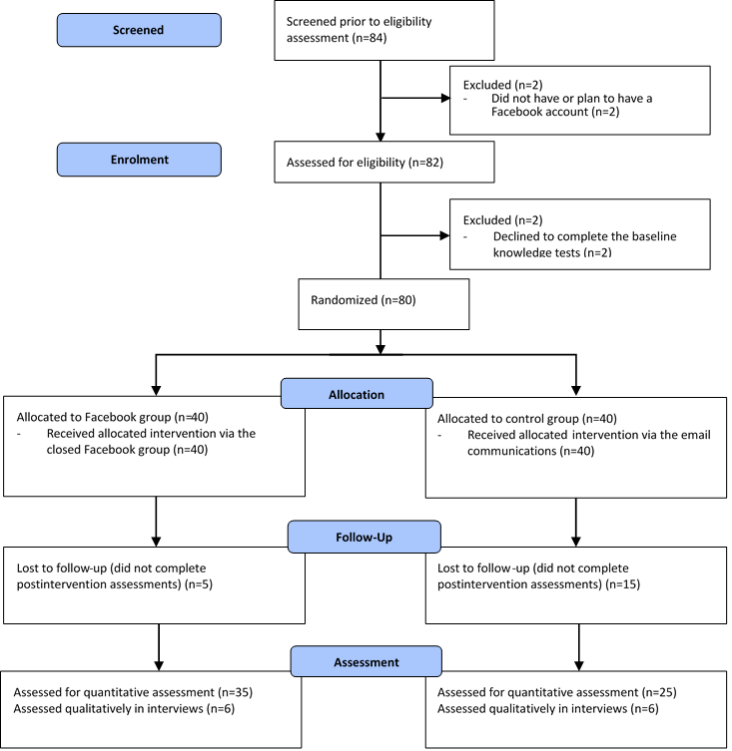
Study CONsolidated Standards of Reporting Trials (CONSORT) flowchart.

Figure 2 shows the flow of study subject recruitment and randomization according to the CONSORT guidelines. Eighty participants underwent randomization with 40 allocated to the intervention group and 40 allocated to the control group.

The overall retention rate was 75% (60/80). Significantly more intervention group participants (35/40, 88%) completed the study compared to those in the control group (25/40, 63%; *P*<.001). Twelve participants, six from each group, attended postintervention interviews.

At baseline, participant characteristics and habits when using SNSs were comparable in the two groups (Table 2 and Multimedia Appendix 1). The majority (64%, 51/80) of participants ranged in age from 20-39 years. The participants, who worked in various health disciplines, included pharmacists, nurses, optometrists, and physiotherapists.

Among all participants, 95% were daily SNS users. WhatsApp Messenger and Facebook were the most frequently used SNSs, followed by Instagram, WeChat, and Line. SNSs were mainly used for personal use and communication as well as for general knowledge. Less than half of the participants had used SNSs for professional communication. Only two participants had previously participated in a CPE program delivered via SNSs (Multimedia Appendix 1).

At baseline, the knowledge test overall mean scores were similar in the two groups (Multimedia Appendix 2). In both groups, the lowest mean correct responses in the DKAS were found in the subscale Communication and Behavior.

**Table 2:** Baseline characteristics of the study participants.

Characteristic			All participants (N=80)		Intervention group (n=40)		Control group (n=40)		*P* value
**Age (years), n (%)**									**.80^a^**
	20-29	28 (35)		14 (35)		14 (35)			
	30-39	23 (29)		13 (33)		10 (25)			
	40-49	15 (19)		6 (15)		9 (23)			
	50-59	11 (14)		5 (13)		6 (15)			
	60-69	3 (4)		2 (5)		1 (3)			
**Sex**									**.60^b^**
	Male	19 (24)		8 (20)		11 (28)			
	Female	61 (76)		32 (80)		29 (73)			
**Profession**									**.70^a^**
	Pharmacist	33 (41)		17 (43)		16 (40)			
	Nurse	30 (38)		16 (40)		14 (35)			
	Others	17 (21)		7 (18)		10 (25)			
	Optometrist	10 (13)		6 (15)		4 (10)			
	Physiotherapist	3 (4)		1 (3)		2 (5)			
	Houseman	1 (1)		0 (0)		1 (3)			
	Occupational therapist	1 (1)		0 (0)		1 (3)			
	Overseas pharmacist and nurse	1 (1)		0 (0)		1 (3)			
	Physician	1 (1)		0 (0)		1 (3)			
**Working experience**									**.67^a^**
	<1 year	3 (4)		0 (0)		3 (8)			
	1-2 years	9 (11)		6 (15)		3 (8)			
	2-3 years	3 (4)		3 (8)		0 (0)			
	3-5 years	10 (13)		4 (10)		6 (15)			
	5-10 years	12 (15)		7 (18)		5 (13)			
	>10 years	43 (54)		20 (50)		23 (58)			

^a^Chi square test.

^b^Fisher exact test.

### Quantitative Evaluation of the Effects of a Facebook Intervention

At the postintervention assessment, intervention group participants demonstrated significant knowledge gains, reflected by significant increases in the total DKAS scores, each of the DKAS subscale scores, and multiple choice questions (Table 3). In the control group, significant knowledge gains were not identified in the DKAS subscales Communication and Behavior and Care Considerations.

A significant difference in changes in the mean knowledge gain scores between the two groups was identified in the DKAS subscale Communication and Behavior, with a medium effect size (Table 3). The CPE program delivered by Facebook was more effective than the control (email provision) at increasing participants’ understanding and knowledge of the communication and behavior of people with dementia. There was no significant difference between the two groups in the total DKAS, multiple choice questions, and other DKAS subscale scores.

The effect size in the change in the overall DKAS score was small. For a full-scale two-armed RCT, assuming a standard error of 5% with 95% power, a total of 272 participants would be required (ie, 136 per group). With attrition rates of 10%, 20%, or 30%, the total number of participants required is 304, 340, and 390, respectively.

**Table 3:** Scores and the comparison of changes in scores at postintervention knowledge assessment.

Assessment	Intervention group (n=40)			Control group (n=40)			95% CI	*P* value	Effect size
	Postintervention score, mean (SD)	Change in score, mean (SD)	*P* value	Postintervention score, mean (SD)	Change in score, mean (SD)	*P* value			
Total DKAS^a^ score (maximum score 50)	36.9 (8.0)	8.3 (8.6)	<.001	35.1 (8.0)	4.9 (8.4)	.007	–1.1-7.8	.13	0.44 (small)
DKAS subscale Causes and Characteristics (maximum score 14)	11.3 (3.0)	2.5 (3.4)	<.001	10.8 (2.5)	1.8 (3.4)	.01	–1.1-2.5	.44	0.20 (small)
DKAS subscale Communication and Behavior (maximum score 12)	7.5 (3.1)	2.1 (3.6)	.001	6.2 (2.6)	0.3 (2.1)	.51	0.4-3.3	.02	0.77 (medium)
DKAS subscale Care Considerations (maximum score 12)	9.5 (2.6)	1.4 (2.8)	.007	9.9 (2.6)	1.1 (2.9)	.07	–1.2-1.8	.70	0.23 (small)
DKAS subscale Risks and Health Promotion (maximum score 12)	8.6 (2.3)	2.3 (3.0)	<.001	8.2 (2.7)	1.7 (3.0)	.009	–1.0-2.2	.48	0.21 (small)
Multiple choice question (maximum score 20)	13.2 (3.9)	4.9 (3.7)	<.001	12.8 (5.0)	3.6 (4.5)	.001	–0.9-3.5	.25	0.26 (small)

^a^DKAS: Dementia Knowledge Assessment Scale.

### Quantitative Evaluation of Participant Compliance

Participant compliance with the educational intervention was significantly higher in the intervention group (mean 6.30, SD 2.28 weeks) than in the control group (mean 3.53, SD 3.21 weeks) (*P*<.001). Overall, 75% of the intervention group participants completed at least 6 weeks or more of the intervention compared to only 35% of the control group participants (Table 4). The mean number of participants accessing the materials was 31.5 (SD 3.9) and 17.6 (SD 5.2) in the intervention and control group, respectively.

Participant compliance was significantly higher in the intervention group (mean 14.9, SD 14.1) than in the control group (mean 2.1, SD 1.8) (*P*<.001) with respect to accessing the 18 external learning resources (9 videos and 9 website links) (Multimedia Appendix 3). Participants who learned via Facebook were significantly more compliant in viewing multimedia content.

**Table 4:** Participant compliance.

Total number of weeks accessed	Intervention group (N=40), n (%)	Control group (N=40), n (%)
0	1 (3)	9 (23)
1	2 (5)	9 (23)
2	1 (3)	1 (3)
3	2 (5)	4 (10)
4	1 (3)	2 (5)
5	3 (8)	1 (3)
6	5 (13)	1 (3)
7	7 (18)	5 (13)
8	18 (45)	8 (20)

### Quantitative Evaluation of Participant Engagement on Facebook

A total of 97 Facebook posts were created for the intervention group. All posts received a considerable number of participant engagement actions (mean 19.03, range 11-33). Polls, which were question-related posts designed to encourage member interaction, attracted the highest degree of participant engagement (mean 20.17), followed by videos (mean 19.56). Links (mean 17.33) generated the lowest number of engagement actions during the intervention period.

### Quantitative Evaluation of Participant Satisfaction With the Educational Intervention

As illustrated by item 10 in the satisfaction evaluation (Table 5), participants were satisfied with the training overall. There was no significant difference between the two groups. Evaluating achievement in each of the four course objectives (items 1-4), the overall mean scores ranged from 3.8 to 3.9 (SD 0.6-0.7). More than 80% of participants regarded the program length, quality, and difficulty level as appropriate.

**Table 5:** Participant satisfaction with the educational intervention.

Item		All (N=60)	Intervention group (n=35)	Control group (n=25)	*P* value
**Achievement of objectives, mean (SD)**					
	1. Describe the prevalence, pathophysiology and recognition of dementia	3.9 (0.6)	3.8 (0.6)	4.0 (0.6)	.28^a^
	2. Apply the key screening and assessment tools for diagnosing dementia	3.8 (0.7)	3.7 (0.7)	3.9 (0.8)	.30^a^
	3. Explain the management and support to both elderly people with dementia and their caregivers	3.9 (0.6)	3.9 (0.6)	4.0 (0.6)	.26^a^
	4. Appraise your role in the delivery of dementia care	3.8 (0.7)	3.7 (0.7)	3.9 (0.7)	.22^a^
	Overall (sum of items 1 to 4)/20	15.4 (2.3)	15.1 (2.3)	15.8 (2.2)	.19^a^
**Learning experiences, mean (SD)**					
	5. The training enhanced my knowledge of dementia care	4.2 (0.6)	4.2 (0.6)	4.1 (0.6)	.75^a^
	6. The learning material was well organized	4.1 (0.7)	3.9 (0.8)	4.2 (0.6)	.18^a^
	7. The learning material was easy to use	3.9 (0.9)	3.8 (1.0)	4.1 (0.8)	.21^a^
	8. I will be able to apply the training information to my professional practice	3.8 (0.8)	3.8 (0.8)	3.8 (0.7)	.89^a^
	9. I would recommend this training to others	4.0 (0.7)	3.9 (0.7)	4.0 (0.7)	.49^a^
	10. Overall, I am satisfied with this training	4.0 (0.6)	4.0 (0.6)	4.0 (0.6)	.81^a^
	Overall (sum of items 1 to 6)/30	23.9 (3.8)	23.6 (4.0)	24.3 (3.5)	.49^a^
**Length of training, n (%)**					**.71^b^**
	About right	49 (82)	27 (77)	22 (88)	
	Too long	10 (17)	7 (20)	3 (12)	
	Too short	1 (2)	1 (3)	0 (0)	
**Quality of information, n (%)**					**.34^b^**
	About right	51 (85)	30 (86)	21 (84)	
	Not enough	4 (7)	1 (3)	3 (12)	
	Too much	5 (8)	4 (11)	1 (4)	
**Level of difficulty, n (%)**					**.45^b^**
	About right	52 (87)	29 (83)	23 (92)	
	Too difficult	8 (13)	6 (17)	2 (8)	
	Too easy	0 (0)	0 (0)	0 (0)	

^a^Independent sample *t* test.

^b^Chi square test.

### Quantitative Evaluation of Participant Attitudes Toward Using Facebook for the CPE Program

Overall, the respondents were positive about using Facebook for a CPE program. Intervention group participants rated the use of Facebook significantly higher (*P*=.03) (Table 6).

Significant differences between the two groups were identified in responses to the survey questions “I would recommend Facebook for other CPE courses” (*P*=.01), “Using Facebook improved my learning experience” (*P*=.02), and “I would be more likely to attend a CPE/CNE course if it used Facebook” (*P*=.02).

At completion of this pilot trial, participants who had learned via Facebook were more willing to use Facebook in future CPE programs.

**Table 6:** Attitudes toward the use of Facebook for continuous professional education programs.

Item	All (N=60), mean (SD)	Intervention group (n=35), mean (SD)	Control group (n=25), mean (SD)	*P* value^a^
1. Using Facebook improved my learning experience	3.6 (0.8)	3.7 (0.9)	3.3 (0.7)	.02
2. Using Facebook helped me stay more engaged	3.5 (0.9)	3.6 (0.9)	3.2 (0.7)	.09
3. Using Facebook enabled me to gain more knowledge	3.4 (0.8)	3.5 (0.9)	3.3 (0.7)	.26
4. Using Facebook will help me apply what I have learned to clinical practice	3.2 (0.9)	3.4 (1.0)	3.0 (0.8)	.09
5. Using Facebook enhanced my education	3.4 (0.9)	3.5 (0.9)	3.2 (0.8)	.28
6. I would be more likely to attend a CPE^b^/CNE^c^ course if it uses Facebook	3.4 (0.9)	3.6 (0.8)	3.1 (0.9)	.02
7. I am likely to use Facebook after the course is over	3.4 (0.9)	3.6 (0.9)	3.1 (1.0)	.05
8. Facebook is easy to use	3.6 (0.8)	3.8 (0.7)	3.4 (0.8)	.05
9. Facebook is intuitive to use	3.5 (0.8)	3.6 (0.8)	3.2 (0.8)	.05
10. I would recommend Facebook for other CPE courses	3.4 (1.0)	3.7 (0.9)	3.0 (0.9)	.01
Overall (sum of items 1 to 10)/50	34.3 (7.5)	36.0 (7.4)	31.8 (6.9)	.03

^a^Independent sample *t* test.

^b^CPE: continuing professional education.

^c^CNE: continuing nursing education.

### Qualitative Assessment of Feasibility and Acceptability

Twenty-nine responses were collected from the feedback forms. Twelve participants, six from the intervention group and six from the control group, attended the semistructured interviews. A wide range of inputs were collected, including comments on current models of dementia care and CPE frameworks. We here focus on the findings that helped in evaluating the design of the study and the feasibility and acceptability of interventions. The qualitative data analyses led to the generation of the following themes.

#### Flexible and Effective Learning via Facebook

Learning through Facebook is highly mobile and flexible, without geographic or time constraints. Most of our participants easily accessed Facebook via the Facebook app installed on their mobile phones. Many stated that they seldom set aside a particular time to study, but rather tended to read posts whenever they had a chance. This was exemplified in the following participant comments: “I am glad that I can learn new knowledge during my daily commute,” and “I appreciate the flexibility provided by Facebook, so I can learn whenever and wherever I want.”

Furthermore, participants appreciated the conciseness and effectiveness of the education. One participant stated, “It’s nice the Facebook posts are kept short, because I can complete them easily.” Facebook provides a user-friendly platform that facilitates information retrieval, particularly during revision. This advantage was exemplified in participant comments such as “I hadn’t expected that posts in Facebook could be organized in such a systematic way,” and “I like the tracking feature in the Facebook group that clearly shows which posts I haven’t read.”

A majority of respondents, including a few from the control group, commented that “More CPE programs should be delivered by Facebook.” A few participants concluded, “The flexibility in learning and attractiveness of the content on Facebook could not be replaced by other platforms.”

#### Motivated and Engaged Participation on Facebook

All respondents from the intervention group appreciated the feeling of being encouraged to learn, which could be attributed to the Facebook automatic notification function. One participants stated “It’s nice to receive notifications of new posts. That reminds me to study.” A few participants liked to finish the new posts promptly after reading the Facebook notifications because it was similar to their usual Facebook habits.

Respondents also valued the sense of community that exists in a CPE course delivered using Facebook. In addition to the learning curriculum designed with a multidisciplinary approach, Facebook participants were also aware of their peers from different health disciplines and they appreciated the opportunity to know how others work in delivering dementia care.

The motivating interactions in the Facebook group also enhanced self-learning. Facebook participants claimed that they did notice how other members react to the posts. Some of them stated, “The polls remind me to take action, particularly when many members have voted.”

#### Enriched Learning With Multimedia Components

The multimedia components in the learning curriculum, such as videos and photos, were well accepted by all respondents, particularly those in the intervention group. They considered that the use of multimedia helped to more comprehensively explain concepts, which increased their motivation to learn. Some participants particularly prefer watching videos over other multimedia sources and considered videos as a good source for deepening their understanding on topics. This was exemplified in the following participant comments: “There should be more videos,” and “Some videos are quite interesting and informative, so I shared them with my friends.”

Participants found that Facebook highly facilitated the viewing of multimedia, which encouraged them to access the materials. Some of them claimed, “I watched most of the videos because they were easily played on the Facebook app.” However, the control group participants might not have utilized the multimedia to the same extent as their intervention group counterparts as described in the following section.

#### Low Compliance in the Control Group

In this study, control group participants generally experienced a less satisfactory learning experience and compliance. Although they might have accessed the materials, the majority did not read them all the way through. All control group respondents agreed that it was straightforward to access the learning materials, and felt that the topics and length were appropriate. Nevertheless, a majority claimed that they were not motivated to study in this one-way eLearning mode. One of the perceived obstacles was time. Control group participants typically felt that they ought to set aside a particular period of time to read through the learning materials. However, in their busy lives they usually failed to do so, and then gave up. This was exemplified by participant comments such as “The topics are interesting, but I feel that I need to set aside a particular time to read the notes. Then, I am too occupied to do so,” and “It seems too heavy and boring to read through all the notes.” Moreover, control group participants tended not to view the multimedia components, as they perceived it to be an inconvenience.

Other than receiving emails from the course administrator, control group participants did not have the chance to interact with other participants. A majority of control group respondents claimed they were not engaged in the course until the moment they were asked to complete the postintervention assessments. A few respondents commented, “I did not feel that I had been enrolled in a course until I was reminded to do the tests.”

#### Technical Issues Encountered in Online Learning

All respondents denied that any particular technical issues hindered their learning.

In both groups, several participants stated a concern that viewing videos on mobile phones would consume too much data. Consequently, they tended to study only when they had a Wi-Fi connection. There were suggestions to enlarge some diagrams and photos to enhance reading on a mobile phone. A few intervention group participants commented that the posts were not easy to read (the font size was quite small) on their mobile phone, and therefore they needed to use a computer.

## Discussion

### Principal Findings

This pilot RCT, as the first to evaluate the effects of using an SNS to deliver a CPE program, demonstrates how a novel educational intervention can result in significant knowledge gains in dementia care to health professionals. The high participant compliance and engagement, together with high satisfaction and acceptability, indicate that Facebook can be an effective and feasible learning tool that could easily be rolled out in practical settings.

### Effectiveness of Facebook in Improving Knowledge

The Facebook intervention succeeded in offering the participants significant knowledge gains in all aspects of the knowledge assessments.

Comparing the means of changes in scores between the two groups, the Facebook intervention excelled at producing significant knowledge gains according to the DKAS subscale Communication and Behavior with a medium effect size. The six items included in this subscale assess how respondents interpret the behavioral symptoms of people with dementia and the proper way to communicate efficiently. Communication is a crucial skill in dementia care, but has been difficult for health personnel to learn [39]. For effective learning, rather than using plain text, all participants were instructed to view a collection of videos, websites, and online resources illustrating actual patient behaviors and communication. Although participants in both groups could access the same resources, significantly more intervention group participants accessed the materials. In addition to attracting participants by showing previews of multimedia contents, Facebook’s mobile app enables users to browse external links within the interface. In contrast, the control group participants tended to skip the links, because opening a separate browser appeared to disrupt their reading. Likewise, respondents in previous studies appraised the key benefit of SNSs as being efficient channels for a complex stream of information and multimedia files with no restriction in terms of location or office hours [40,41].

Facebook includes important features affecting participant performance in an online course, improving instructor effectiveness in facilitating learning [42]. On Facebook, posts were formatted in a concise and appealing way to reduce readers’ cognitive loads. With the Facebook tracking function, participants could review the contents nonsequentially and refer back to materials based on their study preferences. By contrast, in the control group, participants usually perceived reading the learning materials as a heavy workload that hindered their learning. Therefore, Facebook is a more effective learning platform by facilitating the integration of learning into routine habits of daily life.

### Motivated Compliance and Engagement on Facebook

Learning through Facebook encouraged participant engagement and compliance. Indeed, participant retention in the intervention group (88%) was remarkably higher than that in the control group (63%) at study completion. Compared to other Facebook interventions for patients or the public, similarly high retention rates have been noted in studies promoting health-related behavioral changes such as running (78%) [43], physical activity (86%) [44], and weight loss (96%) [45]. The sense of presence and interactions with members in the Facebook group were appreciated by respondents in the evaluation and interviews. A higher degree of engagement could contribute to higher attendance [46], explaining the promise of learning through Facebook. However, the control group participants lacked a sense of belonging and involvement, which may explain the high dropout rate in this group.

Facebook is superior to email communication in providing a learner-centered, instructor-facilitated, more interactive platform for participants, which are demonstrated influencing factors of successful CPE programs [47]. Of note, in many jurisdictions, CPE schemes are voluntary, making it a challenge to motivate health professionals to complete learning programs [48,49]. The main deterrents to participation in a CPE include cost, resources, inability to get time off from work, household responsibilities, and lack of time [48,49]. Facebook features good usability to be an attractive CPE program by providing a learning platform unrestricted by time and geographical factors.

Analyses of intervention group activities were conducted to determine the post type generating the highest participant engagement. Polls attracted the most engagement, followed by videos. Links generated the lowest engagement rates, because they were perceived to lead to an external site, disrupting participants’ reading on Facebook. These findings are consistent with previous studies in the marketing field investigating brand engagement [30,31].

### Participants’ Attitudes Toward Facebook for Professional Education

Participants in this study were found to have positive views toward utilizing Facebook for a CPE program.

Intervention group participants rated their learning experience as significantly improved when using Facebook. They were significantly more supportive of recommending the use of Facebook for other CPE courses. Participants appreciated the convenience, flexibility, and usability offered by Facebook that enables ready participation for health professionals living in different regions, negating geographical boundaries. Likewise, respondents in previous studies appraised the key benefit of SNSs as being efficient channels with no restrictions in terms of location or office hours [40,41].

With the extensive use of SNSs in our daily lives, these positive attitudes toward Facebook highlight the feasibility of conducting further research on SNS use by health professionals.

### Study Strengths

This pilot RCT has several strengths, including the randomized controlled design, learning curriculum developed from a multidisciplinary approach, and evaluation of a novel educational intervention utilizing Facebook with both quantitative and qualitative research approaches. The utilization of validated tools allowed for reliable quantitative measures. The qualitative data analyses enabled gaining deeper understanding of experiences on the educational interventions and identification of factors that were important to the participating health professionals.

### Limitations

There are limitations of this study that warrant mention. Health professionals who had been frequent SNS users were more willing to join this study, which introduced bias in evaluating users’ perceptions of Facebook. A majority of participants were aged between 20 and 39 years. These factors would affect the generalizability of the results to the overall workforce.

Many control group participants dropped out of the study, which made the overall dropout rate (25%) larger than the assumption in estimating the sample size (10%). This may have introduced bias and reduced power, affecting the generalizability, validity, and reliability of the results [50]. This issue is slightly mitigated by the increase in sample size from 60 to 80 and the intention-to-treat approach. However, it is understood that the estimate of effect becomes conservative due to dilution as a result of dropouts [51]. Very few participants took part in the semistructured interviews. Therefore, these responses might not adequately reflect the experiences of all those who completed the study, and response bias was likely.

For practicality, the knowledge tests were self-administered, which reduces the ability to standardize test conditions. Although participants were reminded not to check other resources, it was unfeasible to prevent participants from seeking help from others or checking references.

### Implications

This pilot RCT brings forward several implications for further research and practice.

Facebook was found to offer substantial advantages over conventional online CPE programs, being beneficial to those who prefer distance learning, while maintaining interactions with classmates. Leadership by the administrator and member engagement were identified as crucial factors in SNSs [52]. A larger trial is required to thoroughly examine the potential effectiveness of an SNS-based educational intervention as compared to traditional learning approaches. Given the effect size of 0.44 of this study, the total number of participants required would be 272 for a full-scale two-armed RCT.

The benefits of utilizing multimedia components on Facebook highlight strategies for designing suitable educational content delivered by SNSs. Facebook can be an effective training tool for understanding patients or caregiver communication and behavior. Not restricted to dementia care, Facebook may also be used to deliver training on other mental health conditions such as depression and schizophrenia, or behavioral conditions, including speech and movement disorders.

In future research, in addition to measuring changes in knowledge, the impacts on practical implementation and even patient care would be important to investigate. However, the complexity of behavioral change increases as evaluation of an intervention ascends the hierarchy [53]. For dementia, there are some feasible, validated measurement tools available for this purpose, including attitudes toward dementia and self-confidence in caring for people with dementia [54].

Further research would consider including an economic analysis to investigate the cost of delivering CPE through Facebook compared to other channels such as face-to-face meetings and email communication. One of Facebook’s economic advantages is that the post content can easily be reused. Owing to Facebook’s user-friendly features such as post scheduling, the resources required for program administration are minimal.

### Conclusion

Significantly more intervention group participants completed the study than control group participants (*P*<.001). The high levels of participant compliance and engagement make Facebook a promising method for delivering professional educational programs. Attending a CPE program through Facebook significantly improved participants’ knowledge of dementia (*P*<.001). Compared to the control group, although a significant difference was not observed in the mean of changes in total scores, the Facebook intervention was significantly more effective at improving participants’ knowledge of communication and behavior in dementia patients (*P*=.02). Participants demonstrated positive attitudes toward utilizing Facebook for professional learning. As the first pilot RCT examining the effectiveness of an SNS for professional education for health professionals, the findings of this study provide evidence for the feasibility of using Facebook for intervention delivery in a practical setting.

## Appendix

Multimedia Appendix 1 Participants’ habits in using social network sites at baseline.

Multimedia Appendix 2 Scores in preintervention knowledge tests.

Multimedia Appendix 3 Participant compliance: Number of clicks per external resources.

Multimedia Appendix 4 CONSORT-eHEALTH (V 1.6.1).

## Abbreviations

CONSORT: CONsolidated Standards of Reporting Trials
CPE: Continuous professional education
DKAS: Dementia Knowledge Assessment Scale
eLearning: electronic learning
RCT: randomized controlled trial
SNS: social network site
