# Supplementary material for: Facebook as a Novel Tool for Continuous Professional Education on Dementia: Pilot Randomized Controlled Trial
Source: J Med Internet Res. 2020 Jun 2;22(6):e16772. doi: 10.2196/16772 (PMC7298630; doi:10.2196/16772)
Supplement: Multimedia Appendix 1 [file jmir_v22i6e16772_app1.docx]

**Multimedia Appendix 1. Participants’ habits in using social network sites at baseline**

| **Characteristic** | **All**  **(n=80)** | | **IG**  **(n=40)** | | **CG (n=40)** | | **P-value** |
| --- | --- | --- | --- | --- | --- | --- | --- |
|  | **N** | **%** | **N** | **%** | **N** | **%** |  |
| **Frequency of SNS use** |  |  |  |  |  |  | **1.000**^a^ |
| Daily | 76 | 95.0% | 38 | 95.0% | 38 | 95.0% |  |
| Every 2-3 days | 3 | 3.8% | 2 | 5.0% | 1 | 2.5% |  |
| Weekly | 1 | 1.3% | 0 | 0.0% | 1 | 2.5% |  |
| **Frequently used SNSs ^c^** |  |  |  |  |  |  |  |
| WhatsApp Messenger | 78 | 97.5% | 39 | 97.5% | 39 | 97.5% | 1.000**^b^** |
| Facebook | 74 | 92.5% | 37 | 92.5% | 37 | 92.5% | 1.000**^b^** |
| Instagram | 39 | 48.8% | 22 | 55.0% | 17 | 42.5% | 0.371**^b^** |
| WeChat | 19 | 23.8% | 9 | 22.5% | 10 | 25.0% | 1.000**^b^** |
| Line | 8 | 10.0% | 4 | 10.0% | 4 | 10.0% | 1.000**^b^** |
| LinkedIn | 6 | 7.5% | 2 | 5.0% | 4 | 10.0% | 0.675**^b^** |
| Weibo | 4 | 5.0% | 2 | 5.0% | 2 | 5.0% | 1.000**^b^** |
| Snapchat | 2 | 2.5% | 1 | 2.5% | 1 | 2.5% | 1.000**^b^** |
| Twitter | 1 | 1.3% | 1 | 2.5% | 0 | 0.0% | 1.000**^b^** |
| Yahoo | 1 | 1.3% | 0 | 0.0% | 1 | 2.5% | 1.000**^b^** |
| Google | 1 | 1.3% | 0 | 0.0% | 1 | 2.5% | 1.000**^b^** |
| **Years of using SNSs** |  |  |  |  |  |  | **0.622**^a^ |
| Less than 1 year | 0 | 0.0% | 0 | 0.0% | 0 | 0.0% |  |
| 2 years to less than 3 years | 2 | 2.5% | 1 | 2.5% | 1 | 2.5% |  |
| 3 years to less than 5 years | 4 | 5.0% | 2 | 5.0% | 2 | 5.0% |  |
| 5 years to less than 10 years | 48 | 60.0% | 26 | 65.0% | 22 | 55.0% |  |
| 10 years or above | 26 | 32.5% | 11 | 27.5% | 15 | 37.5% |  |
| **Purpose of using SNSs** ^c,d^ |  |  |  |  |  |  |  |
| Personal use and communication | 79 | 98.7% | 40 | 100.0% | 39 | 97.5% | 1.000**^b^** |
| General knowledge | 48 | 59.5% | 23 | 57.5% | 25 | 62.5% | 0.820**^b^** |
| Professional communication | 34 | 43.0% | 20 | 50.0% | 14 | 35.0% | 0.258**^b^** |
| Attending CPE programme | 2 | 2.5% | 2 | 5.0% | 0 | 0.0% | 0.494**^b^** |
| Seeking information from professional conferences or seminars | 11 | 13.9% | 8 | 20.0% | 3 | 7.5% | 0.193**^b^** |

Note:

^a^: P-value for Chi-square test

^b^: P-value for Fisher’s Exact test

^c^ Respondents could have more than one response.

^d^ Professional communication includes communicating with clients or working partners, making referrals, and discussing professional matters.
