# Supplementary material for: Facebook as a Novel Tool for Continuous Professional Education on Dementia: Pilot Randomized Controlled Trial
Source: J Med Internet Res. 2020 Jun 2;22(6):e16772. doi: 10.2196/16772 (PMC7298630; doi:10.2196/16772)
Supplement: Multimedia Appendix 2 [file jmir_v22i6e16772_app2.docx]

Multimedia Appendix 2. Scores in pre-intervention knowledge tests

| **Assessment** | **IG**  **(n=40)** | | | | **CG**  **(n=40)** | | | | **P-value** |
| --- | --- | --- | --- | --- | --- | --- | --- | --- | --- |
|  | **M** | **SD** | **Min** | **Max** | **M** | **SD** | **Min** | **Max** |  |
| **DKAS** |  |  |  |  |  |  |  |  |  |
| Total/ 50 | 28.1 | 7.8 | 13.0 | 43.0 | 29.9 | 8.1 | 17.0 | 48.0 | 0.323 |
| Causes and characteristics/ 14 | 8.7 | 3.3 | 3.0 | 14.0 | 8.8 | 2.9 | 2.0 | 14.0 | 0.856 |
| Communication and behaviour/ 12 | 5.3 | 2.3 | 1.0 | 12.0 | 5.8 | 2.3 | 2.0 | 10.0 | 0.335 |
| Care considerations/ 12 | 8.0 | 2.5 | 3.0 | 12.0 | 9.0 | 2.6 | 0 | 12.0 | 0.082 |
| Risks and health promotion/ 12 | 6.2 | 2.3 | 1.0 | 11.0 | 6.3 | 2.9 | 1.0 | 12.0 | 0.797 |
| **Multiple choice question/ 20** | 8.4 | 2.1 | 3.0 | 12.0 | 8.6 | 2.8 | 3.0 | 15.0 | 0.686 |

Abbreviations: M, mean; SD, standard deviation; Min, the minimum score; Max, the maximum score.

One sample t test was used in significance analyses.
