# Supplementary material for: Facebook as a Novel Tool for Continuous Professional Education on Dementia: Pilot Randomized Controlled Trial
Source: J Med Internet Res. 2020 Jun 2;22(6):e16772. doi: 10.2196/16772 (PMC7298630; doi:10.2196/16772)
Supplement: Multimedia Appendix 3 [file jmir_v22i6e16772_app3.docx]

Multimedia Appendix 3. Participant compliance: Number of clicks per external resources

| **Week** | **Topic area** | **Type** | **IG**  **(n=40)** | **CG**  **(n=40)** | ***P*-value** |
| --- | --- | --- | --- | --- | --- |
| **1** | Recognition of dementia   - 1. What is dementia?   2. Prevalence and incidence   3. Pathophysiology | Video | 58 | 3 | <.001 |
|  |  | Video | 31 | 3 |  |
|  |  | Website | 31 | 3 |  |
| **2** | Recognition of dementia   - 1. Risk and protective factors   2. Signs and symptoms of dementia | Video | 23 | 7 |  |
|  |  | Video | 21 | 4 |  |
| **3** | Assessment and diagnosis of dementia | Video | 13 | 2 |  |
|  |  | Website | 7 | 1 |  |
|  |  | Website | 5 | 1 |  |
|  |  | Website | 3 | 1 |  |
| **5** | Non-pharmacological management of dementia | Website | 16 | 2 |  |
|  |  | Video | 14 | 4 |  |
|  |  | Website | 14 | 2 |  |
|  |  | Website | 10 | 0 |  |
| **7** | Support to people with dementia and caregivers | Video | 7 | 1 |  |
|  |  | Website | 6 | 0 |  |
|  |  | Video | 5 | 0 |  |
|  |  | Website | 4 | 2 |  |
|  |  | Video^a^ | 0 | 1 |  |

Note:

^a^ Bitly could not record the number of clicks made in the Facebook group, because video was played automatically on Facebook and Bitly could not count the number of clicks. This issue did not occur in the control group, since participants clicked the Bitly link in a PDF document.
